# Honeybee Colony Vibrational Measurements to Highlight the Brood Cycle

**DOI:** 10.1371/journal.pone.0141926

**Published:** 2015-11-18

**Authors:** Martin Bencsik, Yves Le Conte, Maritza Reyes, Maryline Pioz, David Whittaker, Didier Crauser, Noa Simon Delso, Michael I. Newton

**Affiliations:** 1 Nottingham Trent University, NTU, School of Science and Technology, Clifton Lane, Nottingham, NG11 8NS, United Kingdom; 2 Institut National de la Recherche Agronomique, INRA, UR 406 Abeilles et Environnement, Domaine Saint-Paul, CS 40509, 84914, Avignon, France; 3 Centre Apicole de Recherche et d'Information, CARI, 4, Place Croix du Sud, B-1348, Louvain-La-Neuve, Belgium; Arizona State University, UNITED STATES

## Abstract

Insect pollination is of great importance to crop production worldwide and honey bees are amongst its chief facilitators. Because of the decline of managed colonies, the use of sensor technology is growing in popularity and it is of interest to develop new methods which can more accurately and less invasively assess honey bee colony status. Our approach is to use accelerometers to measure vibrations in order to provide information on colony activity and development. The accelerometers provide amplitude and frequency information which is recorded every three minutes and analysed for night time only. Vibrational data were validated by comparison to visual inspection data, particularly the brood development. We show a strong correlation between vibrational amplitude data and the brood cycle in the vicinity of the sensor. We have further explored the minimum data that is required, when frequency information is also included, to accurately predict the current point in the brood cycle. Such a technique should enable beekeepers to reduce the frequency with which visual inspections are required, reducing the stress this places on the colony and saving the beekeeper time.

## Introduction

Insect pollination has been demonstrated to be of tremendous importance to crop production and the survival of wild plants [1,2], and honeybees (*Apis mellifera*) play a major role in ensuring these [3,4]. Because of the decline of managed colonies recently observed in Europe [5] and in the USA [6] and with regard to these important pollinator services provided by bees [7], the health of honeybee populations has been a growing concern amongst scientists, ecologists, farmers and policy makers [8,9].

Monitoring for pests, parasites and diseases, as well as colony strength, is a vital element of successful beekeeping. Brood rearing and colony growth depend (among other things) on the queen's reproductive state (i.e. the number of eggs a queen can potentially lay per day) and because larvae are reared by adults, the size of the worker population [10]. The amount of brood that is reared determines the colony's population size and hence future brood rearing. It is also basic information for the beekeeper to estimate bee colony health.

There are important diseases that can affect both sealed and unsealed brood. They are caused by bacteria, fungi, parasites, pesticides or viruses [11]. European Foulbrood (EFB) and American Foulbrood (AFB) are major bacterial diseases, economically important in honeybees worldwide, and highly infectious. AFB is the most damaging bacterial brood disease. Not only does it kill infected larvae but it is also potentially lethal to infected colonies [12]. EFB is also a severe disease, affecting mainly unsealed brood, killing honeybee larvae usually when they are 4–5 days old [13]. Another disease that can severely affect the brood is Chalkbrood, caused by the fungus *Ascosphaera apis*. It can be recognised by the presence of mummified larvae at the entrance or back end of the hive and the presence of a spotty brood pattern. Even if *A*. *apis* is lethal to individual larvae, it usually does not destroy an entire bee colony. However, it can cause significant losses in terms of both bee numbers and colony productivity [14]. *Varroa destructor* also impacts the brood of colonies as it enters the brood cells just before capping, and further reproduces inside capped cells [15]. In severe infestations, the brood symptoms of *Parasitic Mite Syndrome* often resemble those of EFB and sacbrood [16]. Specific pesticides can also affect honeybee immature phases, larvae, nymphs and pupae [17,18] or predispose brood to diseases [19]. Larvae may be exposed to the chemicals both by contact and/or orally. Well known active substances affecting larvae are insect growth regulators such as fenoxycarb, which is used as a toxic standard in toxicological tests on larvae (OECD 2013). However, other molecules (e.g. fungicides like Captan^TM^, Iprodione, Chlorothalonil or Ziram® and acaricides/insecticides like Fluvalinate, Coumaphos or Chloropyrifos) with different modes of action can also affect brood [20,21].

To assess honeybee colony health, beekeepers must open the hive and visually inspect for the presence of diseases and colony development and strength (bee and brood quantity). However, this intrusive inspection is a source of stress to the colony and is also time consuming. Worker bees can be killed during this invasive assessment, and there is also a risk that the queen will be killed in the process [22].

The use of modern sensor technology to monitor honeybee colony status is growing in popularity [23–25]. There are already devices, such as electronic scales for measuring temporal hive weight changes, but the increase in hive weight can be due to increased pollen and nectar collection by foragers even in times of colony stress, including the presence of diseases [26]. It is therefore of interest to develop new non-invasive methods that can further contribute to assessing colony physiological status. Our approach is to use accelerometers inserted in the central frame of the hives to measure vibrational amplitudes in order to provide information on bee population, activity, and development. To validate vibrational data, visual inspection data of the colonies, particularly the brood development, are reported for correlation. We show that suitable vibrational data processing allows highly sensitive monitoring of the brood cycle in the vicinity of the sensor. We also explore the minimum data that are required, when frequency information is included, to accurately determine the current point in the brood cycle.

## Materials and Methods

Honey comb vibrational measurements were undertaken with accelerometers embedded in the centre of hive's frames, in an apiary consisting of 22 hives.

### Apiaries

One ‘Langstroth’ and 19 'Dadant' hives were set up in a line over approximately 40 meters length in Avignon, France, in March 2014, with permission from INRA (owner of the site). They were managed using standard beekeeping practices and continuously monitored for vibrations until November 2014 apart from short power cuts. Only data from this apiary were used for the numerical discrimination exercise.

Two hives ('British Standard National') were set up in Nottingham, UK, in March 2013, with permission from NTU (owner of the site), and were continuously monitored for vibrations for 13 months apart from short power cuts.

The population status of the colonies in Avignon was evaluated seven times, from March to October, using the ColEval [27] method. This method allows estimation of the percentage of each frame having brood, honey and pollen, as well as the number of worker bees in the colony [27]. Hives were opened once a month and each side of each frame was examined.

### Vibrational measurements

Accelerometers (100mV/g in France and 1000mV/g in UK, Brüel and Kjær (Nærum, Denmark) ref: 4705) were pressed into the honey comb of the centre of the central frame of all monitored hives, to monitor vibrations transverse to the plane of the comb. Small amounts of molten wax were poured on the sensors to avoid direct exposure of metallic parts. Spectra with a bandwidth of 5500Hz and a resolution of 3.125 Hz were averaged for 3 minutes and stored on a hard disc with Brüel and Kjær's PULSE software, on a computer set to reboot itself every day at midnight. Data were stored on files covering one day of data (tailored PULSE macros supplied by Brüel and Kjær). Although occasional breaks in data logging occurred during power cuts, recordings resumed automatically upon reestablishment of power. Four of the twenty colonies in France were additionally monitored with accelerometers secured, externally, to the middle front face of the brood box (S4 to S7 Figs). One of the colonies in the UK was measured with eight accelerometers embedded in the centre of eight contiguous frames.

Longer spectral averaging was explored by using successive averaged spectra. Histograms were obtained with a bin width sometimes tailored to the vibrational average strength of the colony under consideration, and ranging from 0.1x10^-7^ m/s^-2^ to 0.4x10^-7^ m/s^-2^.

Analysis was undertaken using code written in the Matlab® core (at Nottingham Trent University, NTU), with the additional use of the 'statistics toolbox' for Generalised Linear Modelling, using the glmfit function. We used the DFA algorithm implemented in Matlab® with a routine made available to us by Professor Roy Goodacre (Manchester University, UK). The authors own the code that does the two-step discrimination analysis and have made it available at https://github.com/sci3bencsm/brood_cycle_matlab_code/tree/master.

## Results

### Overnight vibrational amplitude distributions

**Fig 1:**
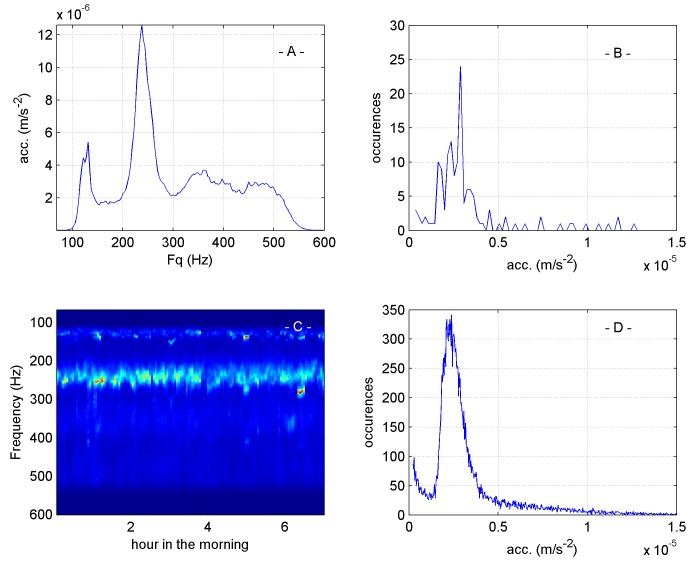
Data processing to obtain the most frequent amplitude in overnight recordings. **(**A) A typical vibrational spectrum averaged over 3 minutes from an accelerometer in the hive. Note the pronounced peaks at 125 Hz and 250 Hz, a feature common to all honeybee vibrational spectra collected from the comb. (B) A histogram of amplitude values from the same data shown in panel A. The majority of amplitudes are found between 1x10^-6^ m/s^-2^ and 5x10^-6^ m/s^-2^, as clearly seen in (A). Note that the information regarding frequency is lost. (C) Vibrational spectra (averaged over 3 minutes) collected from midnight to 7 a.m shown as a spectrogram. Each vertical line is the equivalent of the spectrum in (A) but with the amplitude now colour coded (blue = 0 ms^-2^, red = 3x10^-5^ ms^-2^). The large number of spectra available each night results in the histogram shown in (D) which is much smoother. In subsequent figures all histograms shown have been further normalised to their maximum value, this would be approximately 340 in the case of panel D.

To minimise the effect of daytime foraging activity of the bees and occasional high amplitude spikes in the data set, for example caused by human intervention in the hive, only vibrational frequency spectra measured between midnight and six a.m. were considered. A histogram of the amplitudes was produced as described in Fig 1 and this then lost any frequency information contained within the data.

**Fig 2:**
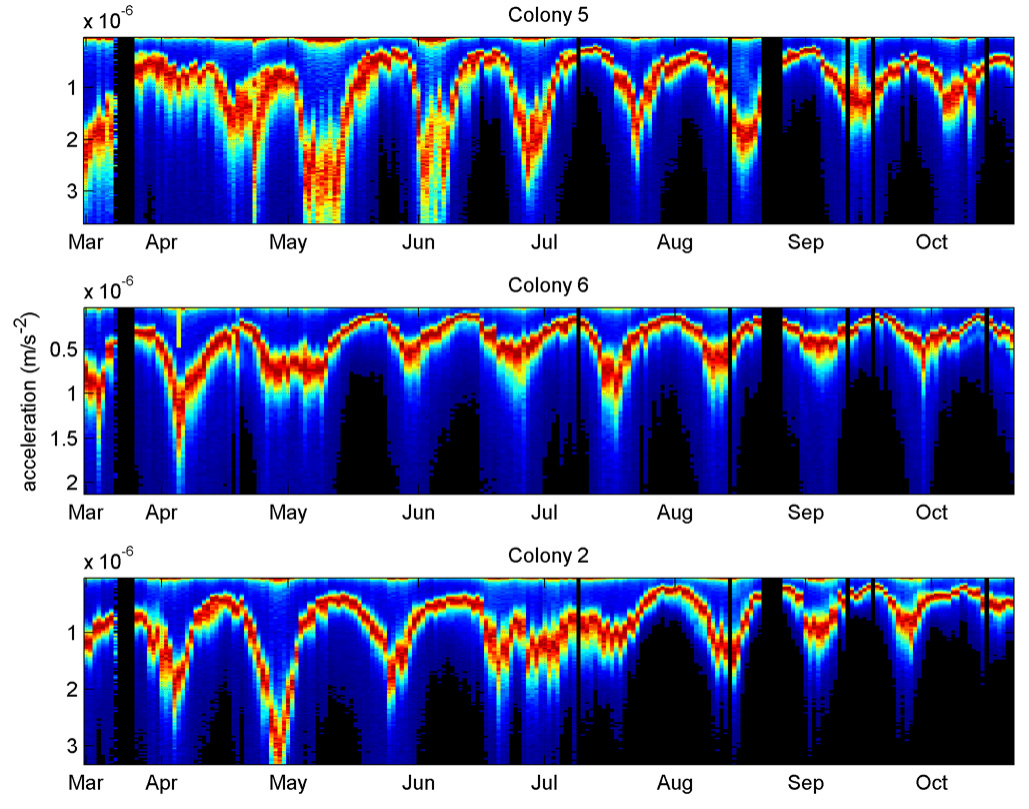
Overnight vibrational amplitude distributions for three colonies. In colony No. 5, the most common vibrational amplitude oscillates regularly over the entire summer, whilst in colony No. 6, a period without a peak in May takes place three weeks after the primary swarm (the date of which is indicated with the yellow tick in early April). Visual inspection revealed that colony No. 2 was temporarily 'drone laying' in July, and this is also reflected as a clear perturbation of the cycle. Histograms are all normalised to their maximum (red pixel). The data for all colonies is available in S1 Fig.

More than one hundred spectra were available each night resulting in a good estimate of the amplitude most often logged during the night. Each histogram was normalised to its maximum value and then colour coded with black equal to zero and red equal to one (the most often occurring amplitude). When a suitable range of vibrational frequencies is considered (see S1 and S2 movies), the histograms exhibit a single, pronounced maximum, which oscillates with a remarkably regular period, closely matched to, although slightly greater than, that of the worker bee brood cycle (Fig 2).

**Fig 3:**
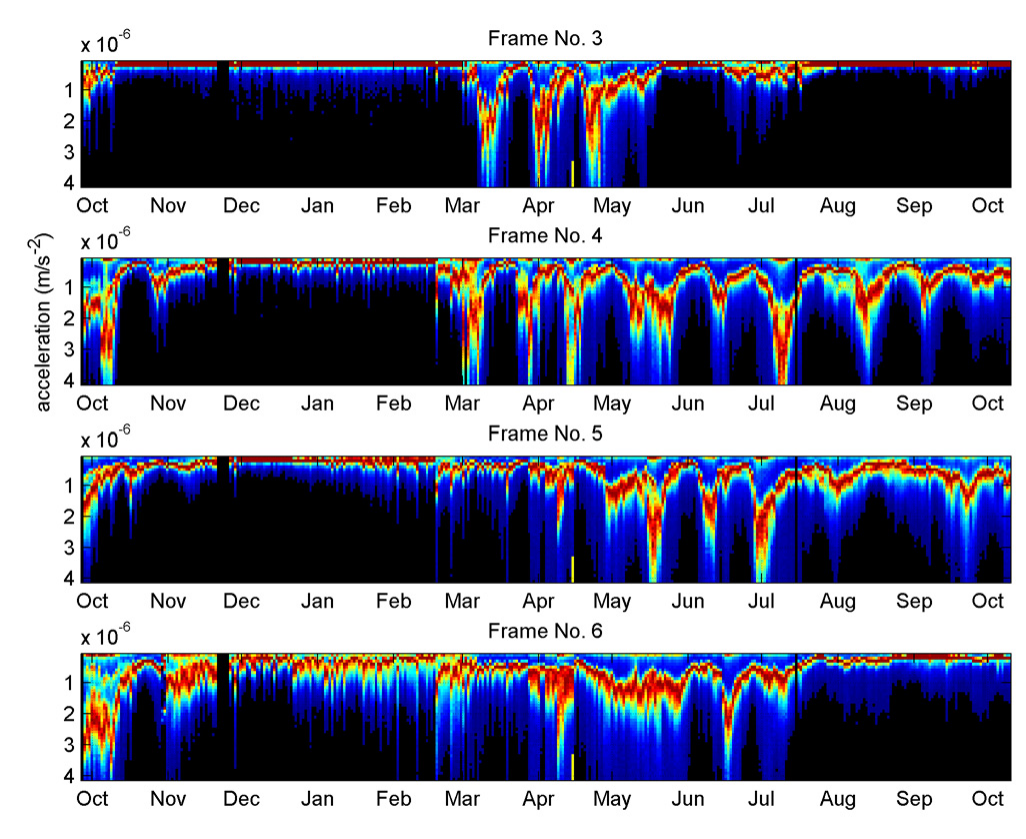
Overnight vibrational amplitude distributions in four contiguous frames within one colony. Note the absence of oscillation in the winter time, the variations in the phase of the oscillation in differing frames, and the lack of oscillation three weeks after the primary swarm in the middle of April (shown by the yellow tick). The data shown here come from the UK apiary.

The regular oscillation is disturbed after a primary swarm or when a colony has lost its queen. It is also disturbed prior to summer colony failure (S1 Fig). It is absent in the winter time and the phase of the oscillation is frame-dependent (Fig 3).

**Fig 4:**
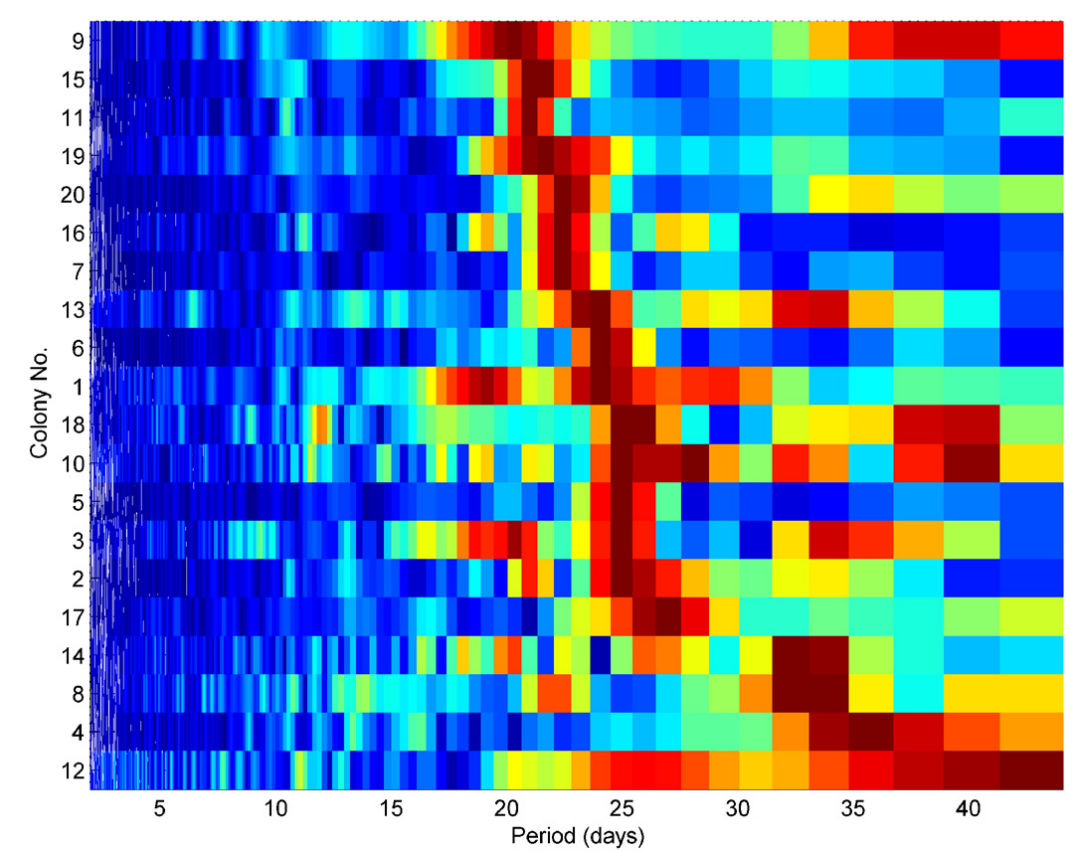
Spectral analysis of the distributions oscillations for 20 colonies of one apiary. All 20 colonies from one apiary are shown, including those used in Fig 2. The spectra have been normalised to their maximum and subsequently sorted to show the colonies with the shortest period (top) to the longest (bottom). Note many spectral maxima in remarkable agreement, between 21 and 26 days. Colony 2, which was found 'drone laying' for a month or so, exhibits small peaks that are additional to the single one seen for Colony 5 and 6. Only one colony (No. 4) out of 20 does not exhibit a clear peak in the range 21 to 26 days. Only four colonies out of 20 do not have their maximum in that same range. Two of these (No. 12, and No. 14) are colonies that failed a month after their primary swarm took place.

The periodically repeating maximum can be extracted, for example by fitting an analytical function to the distributions and the period of the oscillation quantitated by Fourier transformation of the time series of these coordinates. When shown as a function of the period, the spectra exhibit clear maxima between 21 and 26 days (Fig 4).

### Correlation between vibrational measurements and visually assessed frame condition

**Fig 5:**
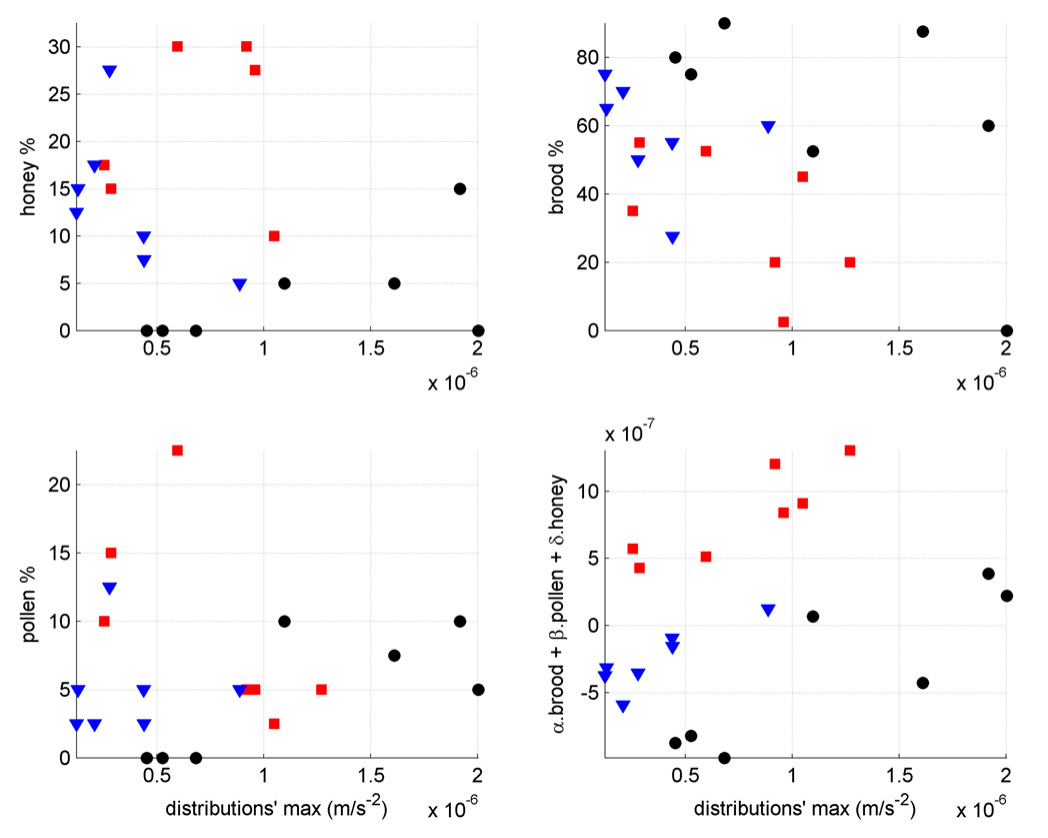
Whole frame condition against vibrational amplitude. Only the three colonies from Fig 2 are considered here (Colony No. 2 is represented by red squares, No. 5 by black circles, and No. 6 by blue triangles), at seven points in time when the frames condition were assessed visually. When colonies are considered individually, the linear combination of frame condition can be correlated to the extracted vibrational signal but the multiplying factors (α, β and δ) are not the same for each frame (red (α, β, δ) = (0.414,0.147,-0.668)x10^-7^, black (α, β, δ) = (0.402,-0.109,0.441)x10^-7^, blue (α, β, δ) = (-0.439,0.0006,0.68)x10^-7^).

The brood, pollen and honey levels visually assessed on most frames exhibit remarkable agreement on either side of the frames. As there is only one sensor in a given frame and it is in the middle, we assume that it is affected equally by each side and the ColEval data were therefore averaged for the frames in which the accelerometer resided. In Fig 5 each quantity is plotted against the maximum in the vibrational amplitude at the seven points in time where visual assessments took place for the three colonies shown in Fig 2.

Pollen levels do not seem to substantially affect the maximum amplitude. Brood and honey levels both contribute to reduce the measured signal and the effect of linearly combining them, together with pollen to further improve the correlation, are also shown in Fig 5; a set of plots for all the hives show a similar correlation and are included in S2 Fig including a linear combination using three generic coefficients, different from those in Fig 5. The contribution to signal damping from the honey levels is the highest even though, on these central frames, honey is seldom greater than 20% of the frame area and is often located at the periphery.

### Single averaged spectrum analysis

**Fig 6:**
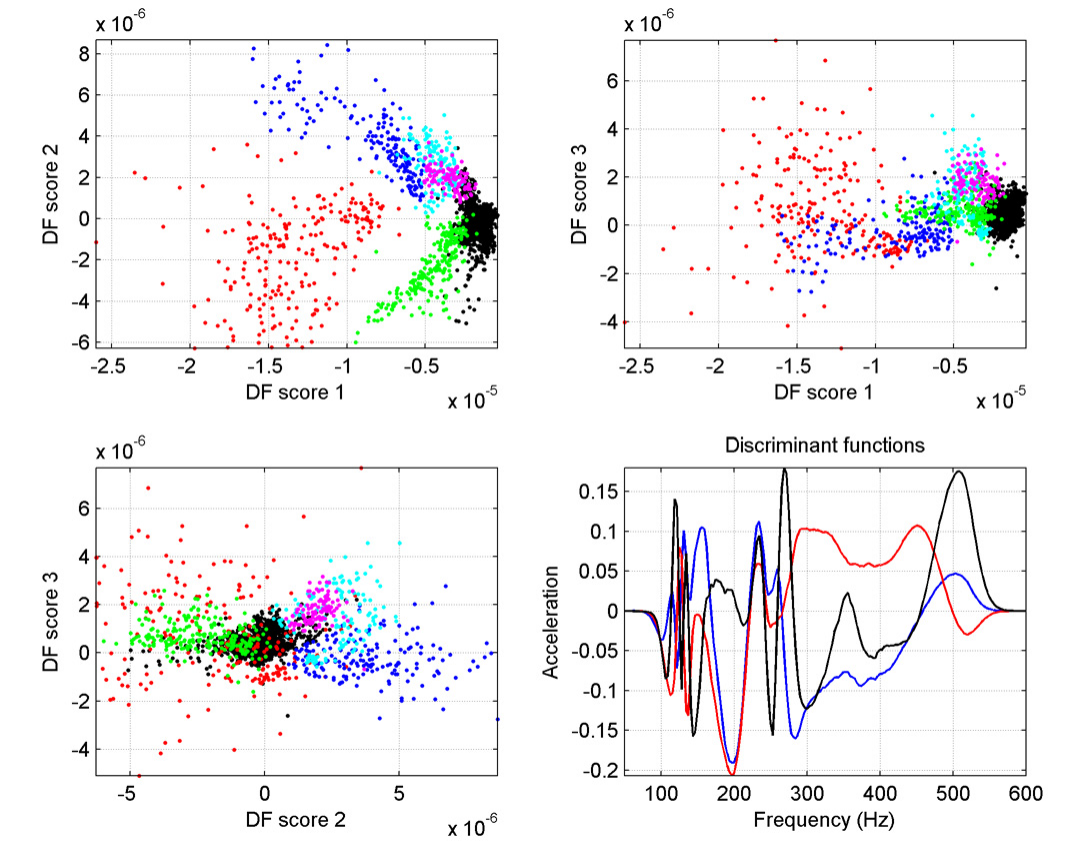
Outcome of numerical discrimination for raw vibrational amplitude spectra. One averaged spectral measurement is collapsed onto a 3D point with coordinates named DF score 1, 2, and 3. Black dots: low amplitude state measurements (any colony 2, 5, 6, 7 or 15). Other dots: high amplitude state measurements for Colony 5 (red), 2 (blue), 6 (green), 7 (cyan) and 15 (magenta). Good discrimination is achieved for 30 minute long measurements except for Colony 6. The last figure gives the discriminant functions, which must be cross-correlated with the mean spectrum to give DF score 1, 2 and 3.

The ability to determine the position in the brood cycle from a single averaged spectrum has been investigated by means of a simple two step clustering exercise; note that we are now including frequency information as well as the amplitude. Five colonies were selected (colonies 2, 5, 6, 7 and 15), on the basis of the clarity and regularity of the oscillations that their distributions exhibit, as candidates to identify an algorithm that would further allow predictive discrimination on the other colonies. Although strict validation of the outcome is not possible (honeycomb loads in the vicinity of the accelerometer are not known), it is useful to explore whether frequency-resolved accelerometer data carries the oscillating information seen in the amplitude data. Spectra were selected at some of the maxima and minima of their respective oscillations, and underwent Principal Component Analysis (PCA) [28]. For each spectrum, the corresponding dominant 10 PCA scores were further fed into a Discriminant Function Analysis (DFA) [29] for supervised discrimination based on troughs and peaks. In order to find discriminant functions with generic effectiveness to all five colonies in the data set, the best results were obtained when the PCA scores were collapsed onto three DFA scores, and when measurements from all five colonies in the 'low vibrational amplitude' state were clustered into a single cloud, whilst measurements in the 'high vibrational amplitude' states were clustered into separate clouds for different colonies. When raw spectra, containing both amplitude and frequency information were fed into the algorithm, suitable clustering could be achieved provided the measurement comprised at least 30 minutes of averaging, as shown in Fig 6.

**Fig 7:**
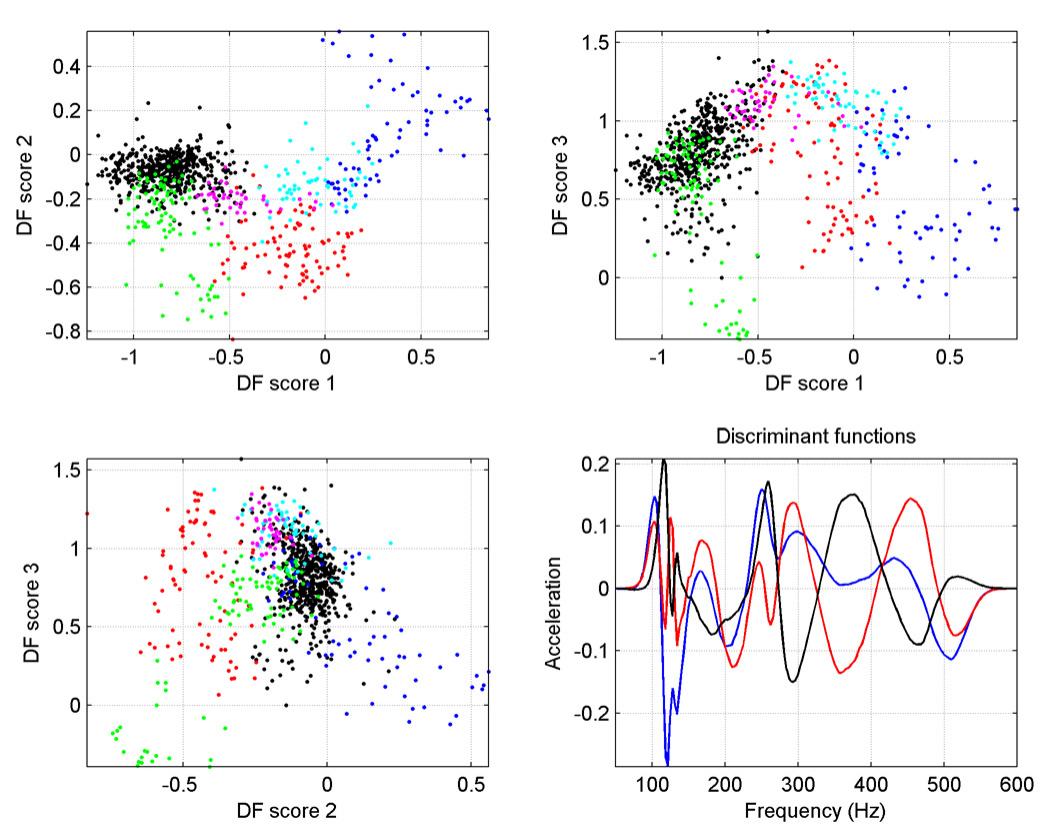
Outcome of numerical discrimination for spectra normalised to their maximum. The colour coding is the same as in Fig 6. Here 60 minute long averaging is required, and Colony 6 still exhibits overlap between the two states being discriminated. The fourth figure gives the relevant discriminant functions.

Clustering is also possible purely on the basis of vibrational spectral shape. However when spectra normalised to their maximum were fed into the algorithm, discrimination necessitates measurements with at least 60 minutes spectral averaging, as can be seen in Fig 7.

The generic discriminant functions identified in Fig 7 are further applied to 60 minute long averaged spectra.

**Fig 8:**
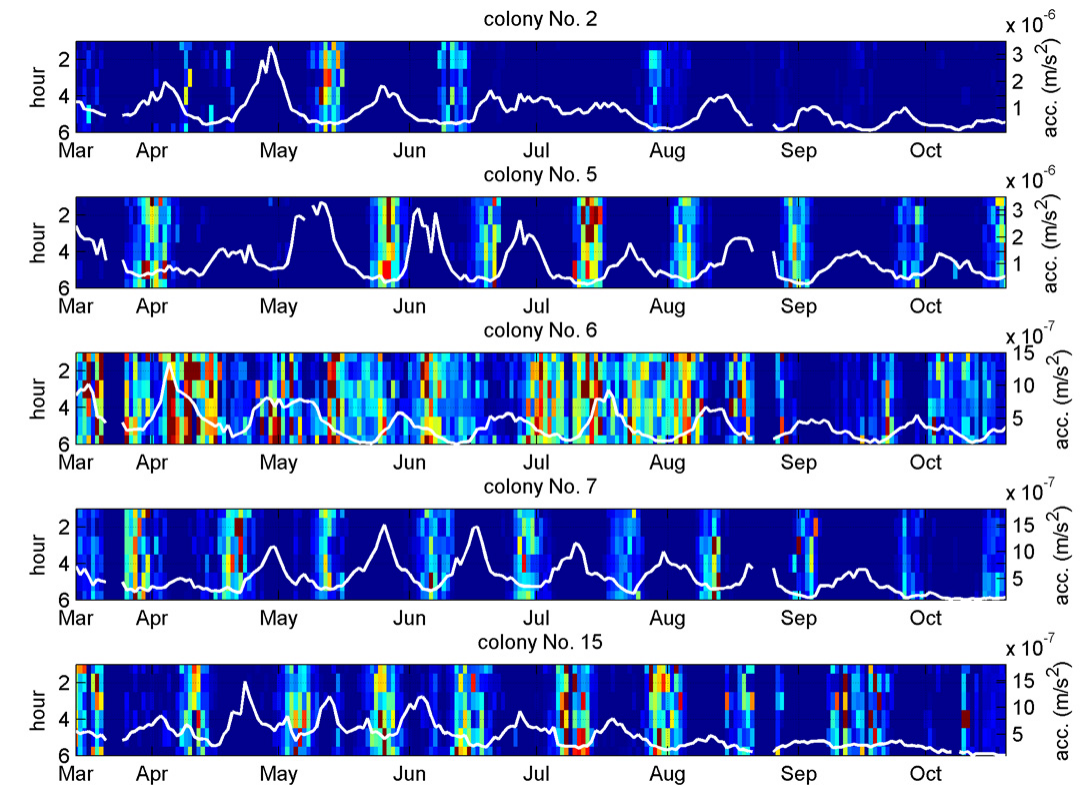
Vibrational amplitude oscillation tracked by spectral shape analysis. One hour long averaged spectra are normalised prior to being cross correlated with the three discriminant functions shown in Fig 7. The outcome is used to compute an indicator of the vibrational amplitude oscillation, which is shown from midnight to 6 am as a colour coded image. The indicator is not only good at tracking the amplitude oscillations (except for Colony No. 6) which is shown with the white curve, it also suffers less drift, as clearly demonstrated on Colony No. 5, 7, and 15. The white curve's quantitative axis is displayed on the right hand side of the individual plots. The indicator is obtained by first collapsing a one hour long averaged spectrum onto a 3D point, by computing three separate cross correlations with the curves shown in Fig 7D. The distance, D_1_, between the 3D point and the centroid of the black cloud in Fig 7 is further calculated, as well as the distance, D_2_, between the 3D point and the centroid of the other clouds. The colour-coded indicator is the ratio D_2_/D_1_.

Using the distance between the resulting 3D point to the centre of the black cloud as the denominator, and that to the centre of the other clouds as the numerator, a ratio purely based on spectral shape is obtained as an indicator of the distribution's oscillation, as shown in Fig 8.

**Fig 9:**
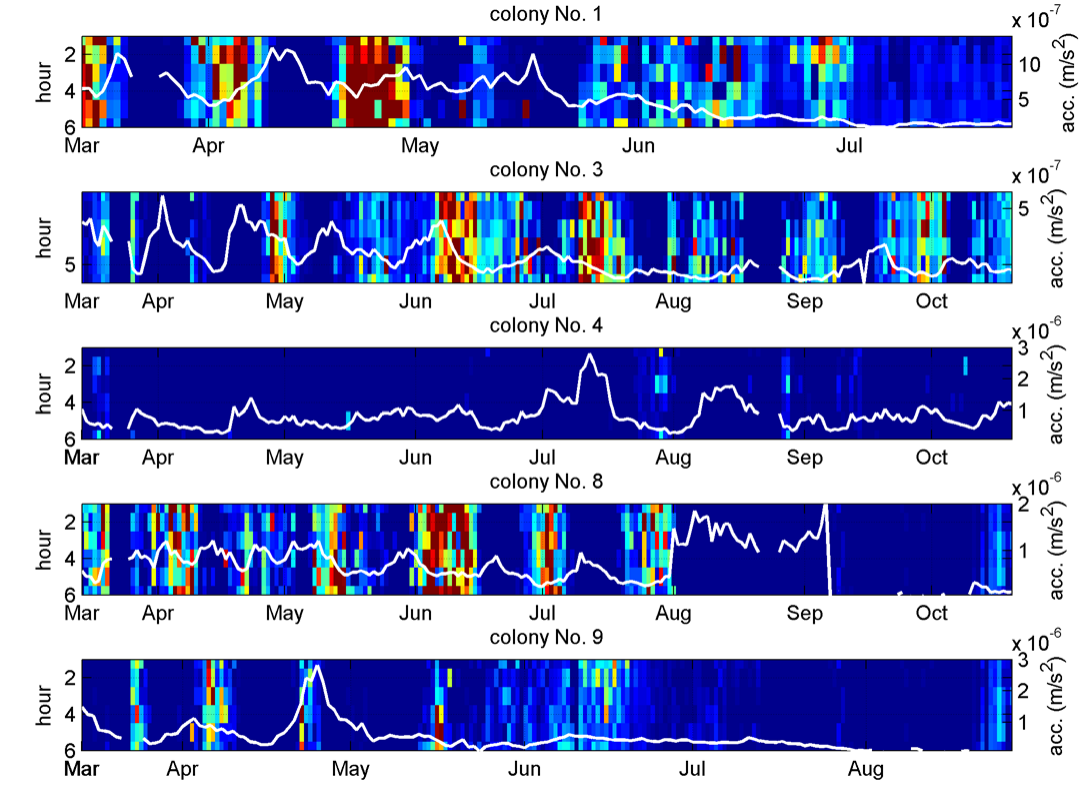
Vibrational amplitude oscillation tracked by spectral shape analysis for colonies that did not contribute to the DFA numerical search. The three discriminant functions shown in Fig 7 are used on the colonies that have not contributed to the DFA numerical search. The resulting predictive indicator is shown again from midnight to 6 am as a colour coded image. The indicator is good at tracking the amplitude oscillations (shown with the white curve) on Colonies 1, 3, 8, 10, 11, 19 and 20, but exhibits poor performance on other colonies, in particular those with very high amplitudes (Colonies 4 and 13) or those where the depth of the oscillation of interest is much less pronounced. The white curve's quantitative axis is displayed on the right hand side of the individual plots. The data for the remaining colonies is shown in S3 Fig.

In spite of poor performance for colony number 6, the indicator demonstrates that an averaged spectral shape feature, common to many colonies and independent from vibrational strength, can be used to track the distribution's oscillations. The predictive ability of the same indicator on the remaining 15 colonies is good on approximately half of them, and shown in Fig 9 and in S3 Fig).

## Discussion

### Evidence suggesting brood cycle sensing

Numerous features of the vibrational amplitude oscillations that we have highlighted directly point to those of the honeybee brood cycle:

- its period is remarkably constant from colony to colony and slightly longer than 21 days, consistent with honeybees known to take some time to maintain a cell immediately after the birth of a worker bee, before the queen would lay a fresh egg in the same cell. This, together with the fact that the queen may be busy on another frame, accounts of the measured period of our oscillation.
- its absence in the winter time is consistent with the fact that the queen does not lay in that season.
- its absence after a primary swarm and in a drone-laying colony is consistent with the fact that these situations respectively correspond to a temporary absence of brood, or absence of locally synchronous brood, in frames.
- its frame-varying phase is consistent with a honeybee queen concentrating on one side of a frame at a time to lay eggs.
- its maxima taking place at low levels of recorded brood is consistent with the fact that an increase in honey comb density enhances the attenuation of the measured vibrations with a constant stimulus, as a consequence of Newton's second law (denser objects are harder to accelerate with the same force).
- its ability to correlate with a spectral shape change is consistent with the frame load affecting the vibrational modes of the honey comb. As an empty honey comb is gradually loaded with pollen, brood and honey, vibrational spectral changes are expected in addition to the simple signal attenuation mentioned above. In the simple driven harmonic oscillator the acceleration is proportional to the square of the frequency.
- its origin lying in the vibrational modes change of the honey comb is further substantiated by the observation that vibrational signals measured in the wall of the hive do not exhibit the oscillation revealed in the honey comb (see S4 to S7 Figs). With time, the walls of the hive will not suffer 'load' or density changes, except perhaps for small moisture increase/decrease with rain/lack of rain, so any signal changes seen there are essentially coming from the variations in the stimulus causing the recorded vibrations: the honeybee sounds and vibrations. The stimulus is clearly not exhibiting the regular, deep oscillation in amplitude recorded on the honeycomb accelerometers.

The honeycomb may be seen as a vibrating substrate, stimulated by sounds and vibrations originating from honeybees in the colony. As the comb content changes, its corresponding transfer function changes too, and in spite of variations in the stimulating signal (evidenced e.g. by the data coming from the wall of the hive) it is the vibrational transmission changes that dominate the modulation of the measured substrate acceleration. The maxima of the amplitude oscillation (appearing as valleys on Figs 2 and 3, and peaks on Figs 8 and 9) most probably correspond to the intervals between brood-rearing (when cells in the vicinity of the sensor are empty). This correspondence is consistent with (i) with the observed unusually long high amplitude sections taking place after a primary swarm or a drone laying colony, (ii) the ColEval data, (iii) the period closely matching that of the brood cycle, and finally (iv) Newton's second law. The relatively disappointing correlation with the recorded brood levels may be attributed to errors arising from (i) recordings relying on visual estimates, (ii) recordings referring to an entire frame, whilst our accelerometers are more sensitive to solid structures closer to them, (iii) some measurements being affected by deviations in the source of the measured vibrations, i.e. activities of the bees themselves. The outcome of the vibrational amplitude spectral shape analysis is encouraging, in suggesting that the brood cycle could be monitored using only one hour of night time measurements, in a way less sensitive to drifts than when using amplitude alone, more generic to multiple colonies, and more specific to the brood cycle. The exploitation of the results by other researchers requires no sophisticated numerical analysis. The discriminant curves shown in the last plots of Figs 6 and 7 simply need cross-correlating with the measured averaged spectrum to give a set of 3D coordinates used to compute the distance to the centroid of interest. Therefore, all that is needed by others is the discriminant curves and the coordinates of the centroid(s) of interest.

Improved brood cycle monitoring could be obtained by applying an artificial stimulus to the frame with known amplitude and frequency rather than relying on the bees to generate the vibrations, and potentially allowing further specific sensitivity to brood, honey and pollen to be identified. Inexpensive accelerometer sensors that cover the relevant range of frequencies are readily available. An improved validation of the signal's sensitivity could be obtained by regular photographic assessment of the vicinity of the accelerometers. This would allow us to check e.g. the hypothesis that queens that lay brood with minimal interleaved empty cells will produce a frame with a vibrational oscillation with a deeper peak-to-trough ratio than those where interleaved empty cells are common.

Mathematical models of the normal brood cycle can easily be performed as a function of the time of the year using vibrational measurements from accelerometer and can be used as a tool to detect abnormal brood cycle to be transmitted to the beekeeper. This information can be helpful to the beekeeper as they can visit the colony to diagnose and solve the problem. Abnormal brood cycle may result from diseases, swarming, queen failure, pesticide exposure or lack of room in the hive. In case of a disease, it can be controlled using medicine or beekeeping techniques or destroying the colony; this would be required in the case of American Foulbrood to avoid the disease spreading to other colonies. This tool also opens new promising opportunities for the testing of toxicological effects at the colony level, enabling long-term, non-invasive observations and bridging the gap between laboratory and field tests. If the abnormal brood cycle is due to swarming, then the beekeeper will have to check for a new queen and follow the development of the colony. Detecting a queen failure will be useful so that the beekeeper can replace it or introduce the workers of the colony to another one with a queen so as to save the queenless workers.

Monitoring of the brood cycle is an interesting scientific tool to measure population dynamics of honeybee colonies. As brood development is closely linked to the climate, it can be performed in different regions of the world or of a country to look at differences in brood development. There are 28 different geographical bee subspecies [30], some of which have a very different brood cycle. Some ecotypes have even been identified to have a brood cycle linked to the blooming of local flowers [31]. Measuring the evolution of the brood cycle of those honeybees is of important evolutionary and ecological interest.

## Supporting Information

S1 Fig Overnight vibrational distributions for all twenty colonies on the INRA apiary. The clear vibrational signal drop suggests a colony failure respectively in early July, early August, and late August for colony 1, 9, and 12. All three colonies generated a primary swarm (indicated with a yellow vertical bar) and several secondary swarms within that summer, and colonies most likely failed from lack of a fertile queen. Note the absence of the periodic cycle prior to failure. Actual colony failures were assessed by visual inspection (which took place once a month). Note the common deterioration of the cycle's depth, on numerous colonies, towards the late summer and early autumn.
(DOCX)

S2 Fig Whole frame condition against vibrational amplitude. All colonies that survived the summer are considered here, at the same seven points in time when frames' condition were assessed visually. All data points are fed into one generic linear combination to obtain the correlation plot shown in the fourth subfigure.
(DOCX)

S3 Fig Vibrational amplitude oscillation tracked by spectral shape analysis for the remaining colonies. The three discriminant functions shown in Fig 7 are used on all colonies that have not contributed to the DFA numerical search. The resulting predictive indicator is shown again from midnight to 6 am as a colour coded image. The indicator is good at tracking the amplitude oscillations (shown with the white curve) on Colonies 1, 3, 8, 10, 11, 19 and 20, but exhibits poor performance on other colonies, in particular those with very high amplitudes (Colonies 4 and 13) or those where the depth of the oscillation of interest is much less pronounced. The white curve's quantitative axis is displayed on the right hand side of the individual plots.
(DOCX)

S4 Fig Overnight vibrational distributions from honey comb and hive wall. The same colony (No 10) is monitored from within the central honey comb (top) and from the middle of the front wall of the brood box (bottom). The vibrational amplitude is typically one order of magnitude lower in the wood, and does not exhibit the regular wave that can be seen from within the honey comb. Similar observations can be made on the three other colonies monitored both from the honey comb and the hive wall, shown in the next three figures.
(DOCX)

S5 Fig Overnight vibrational distributions from honey comb and hive wall. The measurements are those of Colony No 2.
(DOCX)

S6 Fig Overnight vibrational distributions from honey comb and hive wall. This colony (No 20) swarmed in early May, note the substantial loss of signal in the wall, which is not reflected when measured from within the honey comb.
(DOCX)

S7 Fig Overnight vibrational distributions from honey comb and hive wall. The measurements are those of Colony No 16.
(DOCX)

S8 Fig Overnight vibrational distributions from honey comb in UK hives. The four frames, additional to those shown in Fig 3, of the first colony monitored in the UK are shown, as well as the central frame monitored from a separate second UK colony. The yellow tick indicates the primary swarm. Although the 22 days oscillation is not so clear on the peripheral frames, this is expected as the queen often prefers to work towards the centre of the colony. The second colony, monitored from within the central frame, clearly exhibits a pronounced oscillation similar to those highlighted in the manuscript.
(DOCX)

S1 Movie Effect of changing the lower limit of cropped spectra on amplitude distribution. For one colony of interest (colony 6), overnight histograms of amplitude distribution are shown (top figure), when the lower limit of the cropped spectra (bottom figure) is increased from 0 to 410 Hz, whilst the upper limit is kept at 600 Hz. The effect is very small, and the top figure remains remarkably stable, with a single pronounced maximum for all distributions, until the upper limit becomes higher than 350 Hz.
(AVI)

S2 Movie Effect of changing the upper limit of cropped spectra on amplitude distribution. For the same colony (colony 6), overnight histograms of amplitude distribution are shown (top figure), when the upper limit of the cropped spectra (bottom figure) is increased from 255 to 800 Hz, whilst the lower limit is kept at 10 Hz. The effects are large and varied. To start with, most amplitude distributions exhibit two peaks, although some have one or three peaks. As the upper limit becomes larger than 400 Hz, a steady states emerges, gradually leading to extremely well defined unique maxima for each night under investigation. The oscillation of interest in our study is seen with the highest contrast for an upper limit around 600 Hz.
(AVI)
