# Supplementary material for: Honeybee Colony Vibrational Measurements to Highlight the Brood Cycle
Source: PLoS One. 2015 Nov 18;10(11):e0141926. doi: 10.1371/journal.pone.0141926 (PMC4651543; doi:10.1371/journal.pone.0141926)
Supplement: S1 Fig — The clear vibrational signal drop suggests a colony failure respectively in early July, early August, and late August for colony 1, 9, and 12. All three colonies generated a primary swarm (indicated with a yellow vertical bar) and several secondary swarms within that summer, and colonies most likely failed from lack of a fertile queen. Note the absence of the periodic cycle prior to failure. Actual colony failures were assessed by visual inspection (which took place once a month). Note the common deterioration of the cycle's depth, on numerous colonies, towards the late summer and early autumn. (DOCX) [file pone.0141926.s001.docx]

**Figure S1| Overnight vibrational distributions for all twenty colonies on the INRA apiary.** The clear vibrational signal drop suggests a colony failure respectively in early July, early August, and late August for colony 1, 9, and 12. All three colonies generated a primary swarm (indicated with a yellow vertical bar) and several secondary swarms within that summer, and colonies most likely failed from lack of a fertile queen. Note the absence of the periodic cycle prior to failure. Actual colony failures were assessed by visual inspection (which took place once a month). Note the common deterioration of the cycle's depth, on numerous colonies, towards the late summer and early autumn.
