# Supplementary material for: Honeybee Colony Vibrational Measurements to Highlight the Brood Cycle
Source: PLoS One. 2015 Nov 18;10(11):e0141926. doi: 10.1371/journal.pone.0141926 (PMC4651543; doi:10.1371/journal.pone.0141926)
Supplement: S2 Fig — All colonies that survived the summer are considered here, at the same seven points in time when frames' condition were assessed visually. All data points are fed into one generic linear combination to obtain the correlation plot shown in the fourth subfigure. (DOCX) [file pone.0141926.s002.docx]

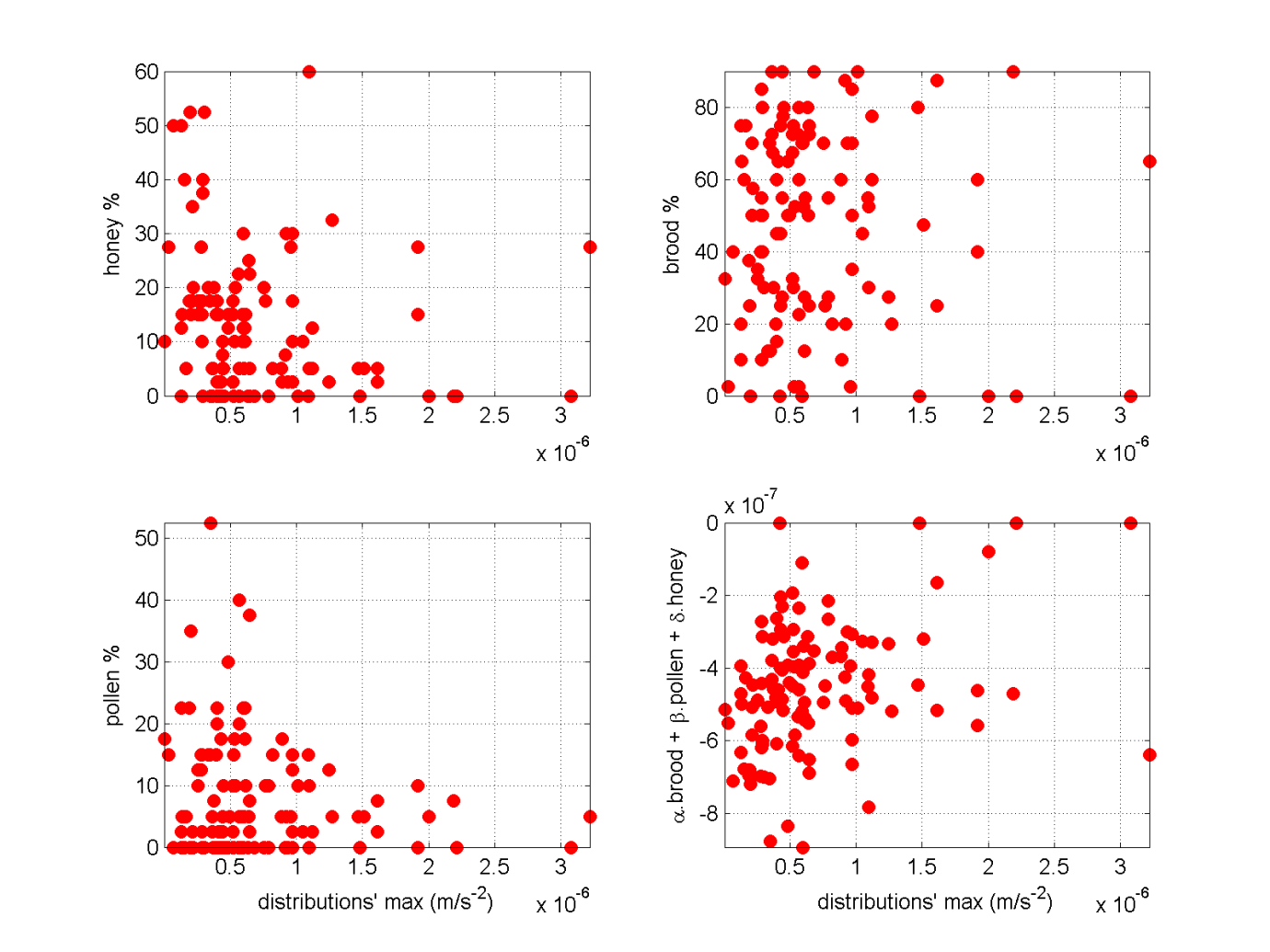


**Figure S2 | Whole frame condition against vibrational amplitude.** All colonies that survived the summer are considered here, at the same seven points in time when frames' condition were assessed visually. All data points are fed into one generic linear combination to obtain the correlation plot shown in the fourth subfigure.
