# Supplementary material for: Honeybee Colony Vibrational Measurements to Highlight the Brood Cycle
Source: PLoS One. 2015 Nov 18;10(11):e0141926. doi: 10.1371/journal.pone.0141926 (PMC4651543; doi:10.1371/journal.pone.0141926)
Supplement: S3 Fig — The three discriminant functions shown in Fig 7 are used on all colonies that have not contributed to the DFA numerical search. The resulting predictive indicator is shown again from midnight to 6 am as a colour coded image. The indicator is good at tracking the amplitude oscillations (shown with the white curve) on Colonies 1, 3, 8, 10, 11, 19 and 20, but exhibits poor performance on other colonies, in particular those with very high amplitudes (Colonies 4 and 13) or those where the depth of the oscillation of interest is much less pronounced. The white curve's quantitative axis is displayed on the right hand side of the individual plots. (DOCX) [file pone.0141926.s003.docx]

**Figure S3 | Vibrational amplitude oscillation tracked by spectral shape analysis for the remaining colonies.** The three discriminant functions shown in Figure 7 are used on all colonies that have not contributed to the DFA numerical search. The resulting predictive indicator is shown again from midnight to 6 am as a colour coded image. The indicator is good at tracking the amplitude oscillations (shown with the white curve) on Colonies 1, 3, 8, 10, 11, 19 and 20, but exhibits poor performance on other colonies, in particular those with very high amplitudes (Colonies 4 and 13) or those where the depth of the oscillation of interest is much less pronounced. The white curve's quantitative axis is displayed on the right hand side of the individual plots.
