# Supplementary material for: Honeybee Colony Vibrational Measurements to Highlight the Brood Cycle
Source: PLoS One. 2015 Nov 18;10(11):e0141926. doi: 10.1371/journal.pone.0141926 (PMC4651543; doi:10.1371/journal.pone.0141926)
Supplement: S4 Fig — The same colony (No 10) is monitored from within the central honey comb (top) and from the middle of the front wall of the brood box (bottom). The vibrational amplitude is typically one order of magnitude lower in the wood, and does not exhibit the regular wave that can be seen from within the honey comb. Similar observations can be made on the three other colonies monitored both from the honey comb and the hive wall, shown in the next three figures. (DOCX) [file pone.0141926.s004.docx]

**

**

**Figure S4 | Overnight vibrational distributions from honey comb and hive wall.** The same colony (No 10) is monitored from within the central honey comb (top) and from the middle of the front wall of the brood box (bottom). The vibrational amplitude is typically one order of magnitude lower in the wood, and does not exhibit the regular wave that can be seen from within the honey comb. Similar observations can be made on the three other colonies monitored both from the honey comb and the hive wall, shown in the next three figures.
