# Supplementary material for: Honeybee Colony Vibrational Measurements to Highlight the Brood Cycle
Source: PLoS One. 2015 Nov 18;10(11):e0141926. doi: 10.1371/journal.pone.0141926 (PMC4651543; doi:10.1371/journal.pone.0141926)
Supplement: S6 Fig — This colony (No 20) swarmed in early May, note the substantial loss of signal in the wall, which is not reflected when measured from within the honey comb. (DOCX) [file pone.0141926.s006.docx]

**Figure S6 | Overnight vibrational distributions from honey comb and hive wall.** This colony (No 20) swarmed in early May, note the substantial loss of signal in the wall, which is not reflected when measured from within the honey comb.
