# Supplementary material for: Honeybee Colony Vibrational Measurements to Highlight the Brood Cycle
Source: PLoS One. 2015 Nov 18;10(11):e0141926. doi: 10.1371/journal.pone.0141926 (PMC4651543; doi:10.1371/journal.pone.0141926)
Supplement: S7 Fig — The measurements are those of Colony No 16. (DOCX) [file pone.0141926.s007.docx]

**Figure S7 | Overnight vibrational distributions from honey comb and hive wall.** The measurements are those of Colony No 16.
