# Supplementary material for: Honeybee Colony Vibrational Measurements to Highlight the Brood Cycle
Source: PLoS One. 2015 Nov 18;10(11):e0141926. doi: 10.1371/journal.pone.0141926 (PMC4651543; doi:10.1371/journal.pone.0141926)
Supplement: S8 Fig — The four frames, additional to those shown in Fig 3, of the first colony monitored in the UK are shown, as well as the central frame monitored from a separate second UK colony. The yellow tick indicates the primary swarm. Although the 22 days oscillation is not so clear on the peripheral frames, this is expected as the queen often prefers to work towards the centre of the colony. The second colony, monitored from within the central frame, clearly exhibits a pronounced oscillation similar to those highlighted in the manuscript. (DOCX) [file pone.0141926.s008.docx]

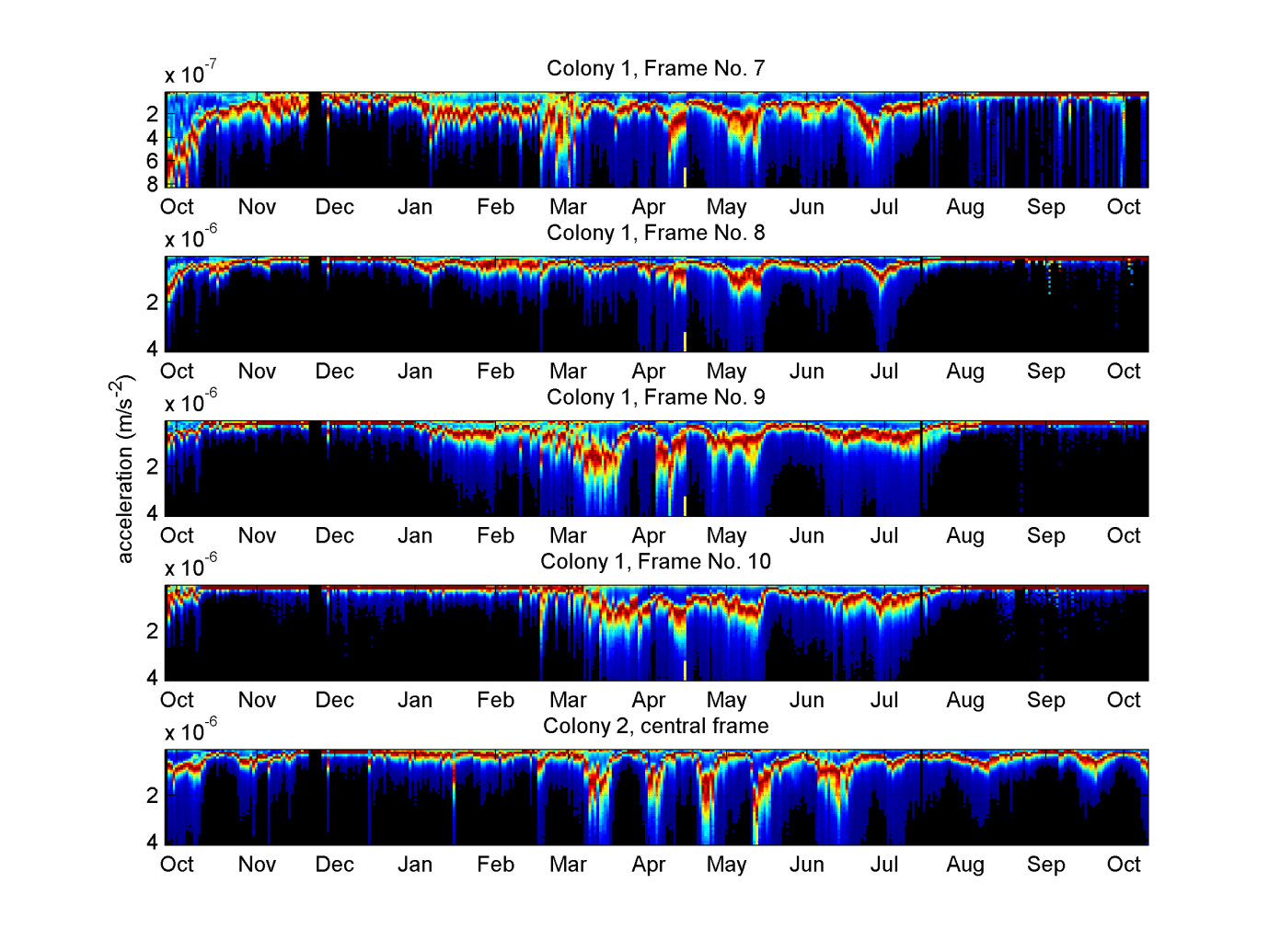


**Figure S8 | Overnight vibrational distributions from honey comb in UK hives.** The four frames, additional to those shown in Figure 3, of the first colony monitored in the UK are shown, as well as the central frame monitored from a separate second UK colony. The yellow tick indicates the primary swarm. Although the 22 days oscillation is not so clear on the peripheral frames, this is expected as the queen often prefers to work towards the centre of the colony. The second colony, monitored from within the central frame, clearly exhibits a pronounced oscillation similar to those highlighted in the manuscript.
